# BIWT: a bioinformatics walkthrough for embedding spatial multiomics in agent-based models for virtual cells

**DOI:** 10.1093/bioinformatics/btaf571

**Published:** 2025-10-16

**Authors:** Daniel R Bergman, Jeanette A I Johnson, Marwa Naji, Max Booth, Heber Lima da Rocha, Atul Deshpande, Dimitrios N Sidiropoulos, Tamara Lopez-Vidal, Randy Heiland, Luciane T Kagohara, Robert A Anders, Lei Zheng, Elizabeth M Jaffee, Genevieve Stein-O’Brien, Paul Macklin, Elana J Fertig

**Affiliations:** Department of Oncology, Sidney Kimmel Comprehensive Cancer Center, Johns Hopkins University, Baltimore, MD, 21218, United States; Convergence Institute, Johns Hopkins University, Baltimore, MD, 21218, United States; Institute for Genome Sciences, University of Maryland School of Medicine, Baltimore, MD, 21201, United States; Greenebaum Comprehensive Cancer Center, University of Maryland School of Medicine, Baltimore, MD, 21201, United States; Institute for Health Computing, University of Maryland School of Medicine, North Bethesda, MD, 20852, United States; Department of Pharmacology & Physiology, University of Maryland School of Medicine, Baltimore, MD, 21201, United States; Department of Oncology, Sidney Kimmel Comprehensive Cancer Center, Johns Hopkins University, Baltimore, MD, 21218, United States; Convergence Institute, Johns Hopkins University, Baltimore, MD, 21218, United States; Institute for Genome Sciences, University of Maryland School of Medicine, Baltimore, MD, 21201, United States; Department of Biology, Johns Hopkins University, Baltimore, MD, 21218, United States; Department of Applied Mathematics and Statistics, Johns Hopkins University, Baltimore, MD, 21218, United States; Department of Oncology, Sidney Kimmel Comprehensive Cancer Center, Johns Hopkins University, Baltimore, MD, 21218, United States; Department of Intelligent Systems Engineering, Indiana University, Bloomington, IN, 47408, United States; Department of Oncology, Sidney Kimmel Comprehensive Cancer Center, Johns Hopkins University, Baltimore, MD, 21218, United States; Convergence Institute, Johns Hopkins University, Baltimore, MD, 21218, United States; Department of Oncology, Sidney Kimmel Comprehensive Cancer Center, Johns Hopkins University, Baltimore, MD, 21218, United States; Convergence Institute, Johns Hopkins University, Baltimore, MD, 21218, United States; Department of Oncology, Sidney Kimmel Comprehensive Cancer Center, Johns Hopkins University, Baltimore, MD, 21218, United States; Department of Intelligent Systems Engineering, Indiana University, Bloomington, IN, 47408, United States; Department of Oncology, Sidney Kimmel Comprehensive Cancer Center, Johns Hopkins University, Baltimore, MD, 21218, United States; Convergence Institute, Johns Hopkins University, Baltimore, MD, 21218, United States; Department of Oncology, Sidney Kimmel Comprehensive Cancer Center, Johns Hopkins University, Baltimore, MD, 21218, United States; Convergence Institute, Johns Hopkins University, Baltimore, MD, 21218, United States; Department of Pathology, Johns Hopkins University, Baltimore, MD, 21231, United States; Department of Oncology, Sidney Kimmel Comprehensive Cancer Center, Johns Hopkins University, Baltimore, MD, 21218, United States; Convergence Institute, Johns Hopkins University, Baltimore, MD, 21218, United States; Department of Oncology, Sidney Kimmel Comprehensive Cancer Center, Johns Hopkins University, Baltimore, MD, 21218, United States; Convergence Institute, Johns Hopkins University, Baltimore, MD, 21218, United States; Department of Oncology, Sidney Kimmel Comprehensive Cancer Center, Johns Hopkins University, Baltimore, MD, 21218, United States; Convergence Institute, Johns Hopkins University, Baltimore, MD, 21218, United States; Department of Neuroscience, Johns Hopkins University, Baltimore, MD, 21205, United States; Department of Human Genetics, Johns Hopkins University, Baltimore, MD, 21218, United States; Department of Intelligent Systems Engineering, Indiana University, Bloomington, IN, 47408, United States; Department of Oncology, Sidney Kimmel Comprehensive Cancer Center, Johns Hopkins University, Baltimore, MD, 21218, United States; Convergence Institute, Johns Hopkins University, Baltimore, MD, 21218, United States; Institute for Genome Sciences, University of Maryland School of Medicine, Baltimore, MD, 21201, United States; Greenebaum Comprehensive Cancer Center, University of Maryland School of Medicine, Baltimore, MD, 21201, United States; Institute for Health Computing, University of Maryland School of Medicine, North Bethesda, MD, 20852, United States; Department of Applied Mathematics and Statistics, Johns Hopkins University, Baltimore, MD, 21218, United States; Department of Biomedical Engineering, Johns Hopkins University, Baltimore, MD, 21218, United States; Department of Medicine, University of Maryland School of Medicine, Baltimore, MD, 21201, United States

## Abstract

**Summary:**

Whereas transcriptomic and spatial profiling offer static snapshots of tissue structure, mechanistic models use biological rules to predict how tissues evolve. We present the BioInformatics WalkThrough (BIWT) software to directly initialize spatial agent-based models from single-cell and spatial molecular data. We demonstrate how initialization strategies affect tumor–immune dynamics and spatial clustering, positioning BIWT as a software suite to generate data-driven virtual cells representing both experimental and clinical contexts.

**Availability and implementation:**

The BIWT software is available at https://github.com/PhysiCell-Tools/PhysiCell-Studio. The sample dataset for running the BIWT is available at https://zenodo.org/records/16365625. The code and instructions for reproducing the use case example is available at https://github.com/drbergman/BIWT-Paper.

## 1 Introduction

Advances in sequencing technologies have revolutionized our understanding of cellular biology, offering unprecedented insights into cell behavior and interactions (Ståhl *et al.* 2016, Marx 2021). Coupled with bioinformatics tools, we can now profile cells in their spatial context (Williams *et al.* 2022), classify distinct cell types (Alquicira-Hernandez *et al.* 2019, Zhang *et al.* 2023), and infer active signaling pathways (Cabello-Aguilar *et al.* 2020, Cherry *et al.* 2021, Jin *et al.* 2025)—providing a more comprehensive view of tissue organization and function. These advances not only illuminate biological structure but also motivate the development of virtual cell models that predict how tissues evolve over time.

Agent-based modeling (ABM) addresses this need by simulating the behavior of individual cells—represented as agents that interact with each other and their environment according to mathematically defined rules (An *et al.* 2009). This rule-based approach enables in silico exploration of complex, multicellular systems over time, but building and calibrating such models typically depends on expert knowledge and experimental data. Incorporating transcriptomic insights can introduce cell-type-specific parameters and pathway activity, enhancing model accuracy, utility, and reproducibility (Arulraj *et al.* 2024). However, even the first step—initializing agent-based models, particularly in space—remains a critical challenge, as different starting conditions can yield dramatically different outcomes. Despite advances in both bioinformatics and modeling frameworks, bridging these domains in a way that is accessible, reproducible, and spatially aware remains an open challenge in computational biology.

Because single-cell and spatial molecular technologies measure the phenotype of individual cells, the data generated from their analyses perfectly mirror the variables tracked in ABM. This close relationship between computational models and high-throughput data makes ABMs primed for initialization from single-cell and spatial molecular data. Here, we present the BioInformatics WalkThrough (BIWT), an open-source tool that integrates bioinformatics analyses into the ABM framework PhysiCell (Ghaffarizadeh *et al.* 2018).

## 2 Results

### 2.1 Implementation

**Figure 1.**
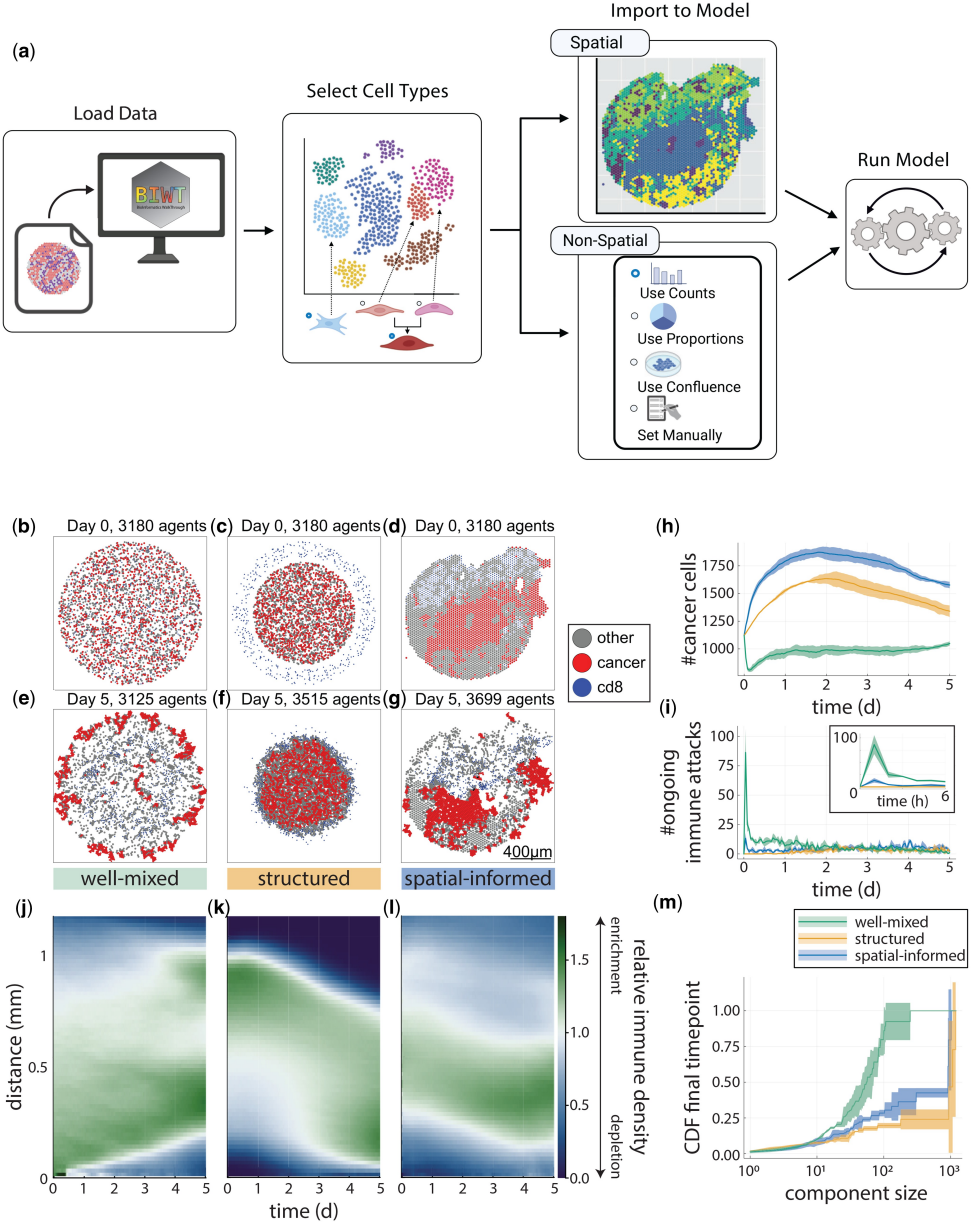
Schematic of the BioInformatics WalkThrough and example use case. (A) Users (1) load their data through a graphical user interface, (2) select the cell types to continue with downstream, (3) import the cells into the model, and (4) run the model. (B–M) The BioInformatics WalkThrough spatial-informed initialization produces distinct dynamics in an agent-based model of tumor-immune interactions. (B–D) Well-mixed (B), structured (C), and spatial-informed (D) initialized models. Legend on the right shows the cell type names used in the PhysiCell model. (E–G) Simulation snapshot at endpoint (*t* = 5 days) for each initialization. (H) Cancer cell population by initialization type over the simulated time. Solid line represents the mean. Shaded area represents ±1 SD. Legend below panel I. (I) Number of ongoing immune attacks at each measured time point. Inset shows these values over the first 6 h. (J–L) Cross-pair correlation function time series heatmaps showing the enrichment or depletion of CD8+ T cells at varying distances from cancer cells. (M) CDF of cancer cell counts in connected components of varying sizes at the final endpoint. Some icons in (A) were generated with Biorender.com.

BIWT provides a Python-based graphical user interface (GUI) that guides users through a streamlined process for converting transcriptomic data into initial conditions for PhysiCell simulations. The workflow proceeds stepwise—from dataset selection and cell type prioritization to data-driven generation of model inputs (Fig. 1; see Methods and Figs S1–S16, available as supplementary data at *Bioinformatics Advances* online for details). BIWT supports commonly used formats from both R and Python pipelines, including Seurat (Hao *et al.* 2021), SingleCellExperiment (Amezquita *et al.* 2020), and AnnData (Wolf *et al.* 2018), as well as CSVs. For non-spatial data, BIWT assigns cells based on their relative abundances in spatial distributions specified by the user. For spatial transcriptomics data, it generates a spatially resolved virtual microenvironment that perfectly matches the spatial distribution of real cells in the measured tissue.

### 2.2 Spatial initialization drives emergent behavior in ABM of tumor-immune dynamics

To illustrate BIWT’s utility, we applied it to an agent-based model of tumor-immune interactions composed of malignant epithelial cells, healthy epithelial cells, and CD8+ T cells (see Methods for details). The model tests how the spatial location of immune infiltration influences tumor progression. Therefore, in the model only cancer cells proliferate or undergo apoptosis; CD8+ T cells migrate and kill cancer cells while healthy epithelial cells neither divide nor die.

We initialized the model using spatial transcriptomics data from a recent clinical trial of neoadjuvant immunotherapies for pancreatic ductal adenocarcinoma (PDAC) (Li *et al.* 2022, Sidiropoulos *et al.* 2024). In all cases, the abundance of each cell type was taken from a surgical biospecimen treated with the triple combination of GVAX, anti-PD1, and CD137 agonist therapies. This sample was selected from the cohort of trial samples as it exhibited high immune cell density at the border of the malignant compartment, making it a compelling test case for BIWT. We compared three initialization strategies of the ABM: a well-mixed initialization, a structured initialization, and a spatial-informed initialization (Fig. 1B–D). In the well-mixed initialization, all three cell types are uniformly distributed in a disc. In the structured initializations, immune cells surround epithelial cells in an annulus. The spatial-informed initialization maps each cell directly from the spatial transcriptomics data, preserving the original coordinates and local microenvironmental structure. Each initialization was simulated three times over five simulated days, yielding nine runs in total (Fig. 1E–G). Supplementary movies (S1–S3, available as supplementary data at *Bioinformatics* online) visualize the progression of each scenario over time.

To assess the impact of BIWT’s spatial-informed initialization on tumor dynamics, we tracked cancer cell counts over time (Fig. 1H). Each initialization produced distinct trajectories. The model with the well-mixed cells had a sharp early decline in cancer cells before stabilizing at 90% of the initial population. The structured and spatial-informed setups followed similar trends but diverged in tumor burden, with the spatial-informed case yielding significantly higher cancer cell counts. Notably, the spatial-informed setup exhibited greater variability in cancer cell counts at early time points across replicates—even though cell placement was identical across runs—whereas the structured initialization, which varied placement, produced more consistent outcomes (Fig. S17, available as supplementary data at *Bioinformatics Advances* online). This counterintuitive result underscores a core property of spatial ABMs: their intrinsic stochasticity enables a single initialization to produce a distribution of outcomes, and this distribution is sensitive to the initial conditions.

We next examined immune activity across the three initialization strategies by tracking ongoing immune cell attacks over time. The well-mixed condition exhibited a sharp, early spike in immune engagement (Fig. 1I), consistent with the initial drop in cancer cell counts observed in this setup (Fig. 1H). Given this relationship, we anticipated lower immune activity in the structured and spatial-informed models, which showed early tumor growth. Nonetheless, the spatial-informed setup exhibited more immune engagements than the structured case, despite faster cancer expansion. Over the full simulation, it also accumulated more immune cell engagements than the structured initialization (Fig. S18, available as supplementary data at *Bioinformatics Advances* online).

To assess how initialization influences immune–tumor colocalization over time, we computed the cross-pair correlation function (cross-PCF) between cancer cells and CD8+ T cells. The cross-PCF quantifies whether T cells are enriched (>1) or depleted (<1) at varying distances from individual cancer cells. We averaged these values across cancer cells at each time point and visualized them as vertical strips in Fig. 1J–L (see Methods, available as supplementary data at *Bioinformatics* online). In the model with well-mixed initialization, immune cells become progressively depleted near cancer cells, likely reflecting successful killing of initially colocalized targets (Fig. 1J). In contrast, the models with structured and spatially informed initializations show progressive immune infiltration, evident in the downward shift of T cell enrichment toward shorter cancer—T cell distances over time (Fig. 1K–L). These divergent patterns illustrate how initialization governs spatial interactions between cancer and immune cells.

Finally, we quantified cancer cell clustering at the final time point to assess how initial conditions shaped tumor architecture. Endpoint snapshots (Fig. 1E–G) reveal visually distinct cancer cell groupings. We quantified these using connected components derived from neighbor graphs in the PhysiCell output (see Methods; Fig. S19, available as supplementary data at *Bioinformatics Advances* online). We then computed the cumulative distribution function (CDF) of cancer cell counts per component, averaging across replicates and shading 1 SD from the mean (Fig. 1M). In the well-mixed initialization, nearly all cancer cells end in small clusters (≤100 cells). In contrast, more than half of the cancer cells in the structured model form large clusters (∼1000 cells). The spatially informed models show an intermediate pattern: most cells belong to large clusters, but around 40% remain in smaller ones. These differences reinforce the importance of initialization in shaping emergent tissue architecture and demonstrate the benefit of using real spatial data to initialize ABMs.

## 3 Conclusion

Integrating biological data directly into mathematical models is a longstanding goal in agent-based modeling (Lorenzo *et al.* 2024). BIWT advances this aim within the PhysiCell framework by enabling users to build models through a graphical interface. By streamlining the creation of data-driven initial conditions, BIWT allows researchers to explore patient-specific dynamics in a reproducible and accessible way (Mangul *et al.* 2019). Our results show that spatial dynamics—such as immune infiltration, tumor compactness, and cell clustering—can significantly influence simulated outcomes. These findings make clear that initialization is not a minor technical detail, but a central design variable that shapes model behavior and interpretability.

Specifically, our simulations represent the competition of tumor cells for limited resources, modeled here as physical space. As a result, the initial spacing of cancer cells has a strong influence on subsequent growth. Over five simulated days, these initialization effects remain measurable and could lead to conflicting interpretations in downstream analyses if not explicitly addressed. BIWT mitigates this risk by grounding initialization in real spatial data, reducing ambiguity and modeler bias in simulations aimed at digital twin applications. We expect that the increased variability observed in simulations initialized from real spatial distributions reflects biological uncertainty in patient outcomes. This underscores the need for future work in uncertainty quantification and in identifying which additional measurements—such as spatial patterns at later time points—would most improve model predictivity. Extending such studies from individual patients to full cohorts will also require reproducible pipelines that support scalable data integration and simulation setup across multiple samples.

BIWT’s modular architecture supports integration with a wide range of data types and modeling frameworks. On the input side, it accommodates standard formats from both R and Python single-cell analysis workflows, ensuring compatibility with evolving data pipelines. On the output side, while BIWT currently targets the PhysiCell framework, its design enables adaptation to other agent-based modeling platforms such as Chaste (Mirams *et al.* 2013) and CompuCell3D (Swat *et al.* 2012), which support distinct mathematical formalisms suited to different biological questions. This interoperability positions BIWT as a flexible bridge between molecular measurements and mechanistic modeling. While BIWT enables model initialization by cell type, future work will extend this platform to automate the assignment of phenotype-specific parameters such as proliferation rate, receptor expression, or metabolic state from atlas studies to enable context-specific model design.

The BioInformatics WalkThrough (BIWT) is the first tool of its kind to bridge bioinformatics pipelines and agent-based modeling through an intuitive, GUI-driven interface. By anchoring spatial agent-based simulations in real molecular data, BIWT supports reproducibility, accessibility, and biological realism. As agent-based models evolve into predictive digital twins for translational research, BIWT provides the infrastructure to transform static molecular profiles into dynamic, interpretable, and patient-specific simulations.

## Supplementary Material

btaf571_Supplementary_Data

## References

[btaf571-B1] Alquicira-Hernandez J , Sathe A, Ji HP et al scpred: accurate supervised method for cell-type classification from single-cell rna-seq data. Genome Biol 2019;20:264. 31829268 10.1186/s13059-019-1862-5 PMC6907144

[btaf571-B2] Amezquita RA , Lun ATL, Becht E et al Orchestrating single-cell analysis with bioconductor. Nat Methods 2020;17:137–45. 31792435 10.1038/s41592-019-0654-x PMC7358058

[btaf571-B3] An G , Mi Q, Dutta-Moscato J et al Agent-based models in translational systems biology. Wiley Interdiscip Rev Syst Biol Med 2009;1:159–71. 20835989 10.1002/wsbm.45 PMC3640333

[btaf571-B4] Arulraj T , Wang H, Ippolito A et al Leveraging multi-omics data to empower quantitative systems pharmacology in immuno-oncology. Brief Bioinform 2024;25:bbae131. 38557676 10.1093/bib/bbae131 PMC10982948

[btaf571-B5] Cabello-Aguilar S , Alame M, Kon-Sun-Tack F et al Singlecellsignalr: inference of intercellular networks from single-cell transcriptomics. Nucleic Acids Res 2020;48:e55. 32196115 10.1093/nar/gkaa183 PMC7261168

[btaf571-B6] Cherry C , Maestas DR, Han J et al Computational reconstruction of the signalling networks surrounding implanted biomaterials from single-cell transcriptomics. Nat Biomed Eng 2021;5:1228–38. 34341534 10.1038/s41551-021-00770-5 PMC9894531

[btaf571-B7] Ghaffarizadeh A , Heiland R, Friedman SH et al Physicell: an open source physics-based cell simulator for 3-d multicellular systems. PLoS Comput Biol 2018;14:e1005991. 29474446 10.1371/journal.pcbi.1005991 PMC5841829

[btaf571-B8] Hao Y , Hao S, Andersen-Nissen E et al Integrated analysis of multimodal single-cell data. Cell 2021;184:3573–87.e29. 34062119 10.1016/j.cell.2021.04.048 PMC8238499

[btaf571-B9] Jin S , Plikus MV, Nie Q. Cellchat for systematic analysis of cell–cell communication from single-cell transcriptomics. Nat Protoc 2025;20:180–219. 39289562 10.1038/s41596-024-01045-4

[btaf571-B10] Li K , Tandurella JA, Gai J et al Multi-omic analyses of changes in the tumor microenvironment of pancreatic adenocarcinoma following neoadjuvant treatment with anti-pd-1 therapy. Cancer Cell 2022;40:1374–91.e7. 36306792 10.1016/j.ccell.2022.10.001 PMC9669212

[btaf571-B11] Lorenzo G , Ahmed SR, Hormuth DA II, et al Patient-specific, mechanistic models of tumor growth incorporating artificial intelligence and big data. Annu Rev Biomed Eng 2024;26:529–60. 38594947 10.1146/annurev-bioeng-081623-025834

[btaf571-B12] Mangul S , Mosqueiro T, Abdill RJ et al Challenges and recommendations to improve the installability and archival stability of omics computational tools. PLoS Biol 2019;17:e3000333. 31220077 10.1371/journal.pbio.3000333 PMC6605654

[btaf571-B13] Marx V. Method of the year: spatially resolved transcriptomics. Nat Methods 2021;18:9–14. 33408395 10.1038/s41592-020-01033-y

[btaf571-B14] Mirams GR , Arthurs CJ, Bernabeu MO et al Chaste: an open source C++ library for computational physiology and biology. PLoS Comput Biol 2013;9:e1002970. 23516352 10.1371/journal.pcbi.1002970 PMC3597547

[btaf571-B15] Sidiropoulos DN , Shin SM, Wetzel M et al Spatial multi-omics reveal intratumoral humoral immunity niches associated with tertiary lymphoid structures in pancreatic cancer immunotherapy pathologic responders. bioRxiv, 2024, preprint: not peer reviewed.

[btaf571-B16] Ståhl PL , Salmén F, Vickovic S et al Visualization and analysis of gene expression in tissue sections by spatial transcriptomics. Science 2016;353:78–82. 27365449 10.1126/science.aaf2403

[btaf571-B17] Swat MH , Thomas GL, Belmonte JM et al Multi-scale modeling of tissues using compucell3d. Methods Cell Biol 2012:110:325–66. 22482955 10.1016/B978-0-12-388403-9.00013-8 PMC3612985

[btaf571-B18] Williams CG , Lee HJ, Asatsuma T et al An introduction to spatial transcriptomics for biomedical research. Genome Med 2022;14:68. 35761361 10.1186/s13073-022-01075-1 PMC9238181

[btaf571-B19] Wolf FA , Angerer P, Theis FJ. Scanpy: large-scale single-cell gene expression data analysis. Genome Biol 2018;19:15. 29409532 10.1186/s13059-017-1382-0 PMC5802054

[btaf571-B20] Zhang S , Li X, Lin J et al Review of single-cell RNA-seq data clustering for cell-type identification and characterization. RNA 2023;29:517–30. 36737104 10.1261/rna.078965.121 PMC10158997

