# Supplementary material for: BIWT: a bioinformatics walkthrough for embedding spatial multiomics in agent-based models for virtual cells
Source: Bioinformatics. 2025 Oct 16;41(11):btaf571. doi: 10.1093/bioinformatics/btaf571 (PMC12597895; doi:10.1093/bioinformatics/btaf571)
Supplement: btaf571_Supplementary_Data [file btaf571_supplementary_data.pdf]

Supplementary Material

Methods

BioInformatics WalkThrough (BIWT)

The BioInformatics WalkThrough is implemented within PhysiCell-Studio (hereafter referred to as Studio), a graphical user interface for building PhysiCell agent-based models (ABMs). Installation instructions for Studio are available in the linked GitHub repository. In addition to Studio's base dependencies, users must install the `anndata` package. For working with R-based data, the `rpy2` and `anndata2ri` packages are also required.

Set up a Python virtual environment, install dependencies, and launch the BIWT interface as follows:

Listing 1. Install dependencies in a dedicated Python environment.

```
python3 -m venv ~/envs/physicell-studio
source ~/envs/physicell-studio/bin/activate
pip install -r PhysiCell-Studio/requirements.txt
```

Then, with the virtual environment activated and in the PhysiCell Studio folder, launch PhysiCell Studio with the –biwt flag:

Listing 2. Launch the BIWT interface.

```
cd PhysiCell-Studio
python bin/studio.py --biwt
```

Users begin by selecting the "ICs" tab from the top-level menu and choosing "BIWT" from the submenu. They are prompted to load a dataset–currently supporting `.h5ad`, `.csv`, or `.rds` formats–and to specify the column containing cell type annotations (Fig. S1). For `.h5ad` files, spatial coordinates are extracted from the obsm field when available. For `.rds` files, spatial coordinates are inferred from coordinate columns in the metadata. In `.csv` files, spatial coordinates are in columns with headers x, y, and (optionally) z. If spatial metadata are detected, users are asked to confirm whether spatial context should be used (Fig. S2).

Dimensionality reduction plots (UMAP, PCA, t-SNE, or spatial plots) are then generated using precomputed results stored in the input data file. These plots help users assess which cell types to retain, merge, or remove (Fig. S3). Users may also rename cell types as desired for representation in PhysiCell (Fig. S4).

When spatial coordinates are not present in the dataset, the user is prompted to specify how many cells of each type to include in the model. This can be done in one of four ways: (1) by using the actual cell counts from the data (Fig. S5), (2) by using cell type proportions to scale the total number of cells (Fig. S6), (3) by setting a desired confluence percentage (Fig. S7), or (4) by manually specifying counts for each cell type (Fig. S8). In this context, confluence refers to the proportion of the 2D microenvironment area that is covered by the cross-sectional area of all cells, analogous to how confluence is estimated in cell culture. When using the proportion-based or confluence-based methods, the values for all cell types are interdependent: adjusting the count, proportion, or confluence of one cell type will automatically update the others to maintain their relative proportions across the dataset. Once cell numbers are finalized through one of these approaches, the user proceeds to the spatial placement step.

Regardless of whether the user begins with spatial or non-spatial data, the next step involves placing cells within the microenvironment. For spatial data, cells are automatically positioned based on their original coordinates, preserving the aspect ratio of the input while scaling and translating it to fit the microenvironment (Fig. S9, S10). Users can further adjust this placement interactively. For non-spatial data, several plotter tools are available to define where cells should be placed: options include "Everywhere" (Fig. S11), "Rectangle" (Fig. S12), "Disc" (Fig. S13), "Annulus" (Fig. S14), and "Wedge" (Fig. S15) plotters, offering flexible strategies for placing cells in biologically or experimentally relevant patterns. These plotters enforce the domain boundaries, ensuring all cells are placed inside the domain even when the shaded region is partially outside the domain.

In both workflows, users can manipulate placement using a combination of mouse and keyboard controls to define regions and fine-tune cell locations. A history feature supports undo and redo actions, allowing users to iteratively adjust placements without losing progress. Parameters for each region can also be entered manually in the interface for precise control. Additionally, a detachable legend window helps users track which colors correspond to which cell types in the plot, enhancing interpretability while designing the initialization.

Finally, users specify an output folder and filename, with the option to either overwrite an existing file or append to a previously saved dataset (Fig. S16). The resulting output is a well-structured CSV file containing the cell positions and types, ready to be integrated into PhysiCell.

Agent-based model

The `cancer` cell type represents malignant epithelial cells that proliferate and spontaneously apoptose; the `cd8` cell type represents CD8+ T cells that follow chemotactic gradients towards apoptosing epithelial cells and attack living malignant epithelial cells encountered along the way triggering their apoptosis; and the `other` cell type represents healthy epithelial cells that occupy the remaining space neither proliferating nor undergoing apoptosis for simplicity of the model. The model was run with PhysiCell v1.14.2. Several project folders are provided in the online materials as well as instructions on how to run them.

Spatial data and model initializations

Spatial transcriptomics (ST) data is obtained from surgical resections of pancreatic cancer described elsewhere [Sidiropoulos et al., 2024]. Cell types were estimated from the ST data to annotate CD8+ T cells, cancer cells, and other cells. Since the ST data is spot-based, the maximum likelihood estimate of the cell type was used to place cells in each spot. Using the cell counts from the ST, the well-mixed and structured model setups were created to match the size of the ST data.

Pair correlation function (PCF)

The pair correlation function (PCF) computes the density of (target) points from a given set in annular regions centered on (center) points from a second, possibly identical, set. If these two sets are the same, it is called PCF. If they are different sets, it is called cross-PCF. These densities are then normalized by the average density of the target set across the area. Thus, values less than 1 in a given annular region indicate depletion of the target points at that distance from the center points, and values greater than 1 indicate enrichment.

We computed the cross-PCF of (target) CD8+ T cells relative to (center) cancer cells to quantify the average proximity of immune cells to cancer cells and thus how likely cancer cells are to be cleared. Thus, for each cancer cell indexed by i, x_i, we first compute $\mathbf{c}_i$ as the vector of immune cell counts at varying distances from x_i at concentric, non-overlapping annuli. In the following formula, let $\mathcal{I}$ be the set of all immune cells and $d(x, y)$ be the distance between two cells.

$$\mathbf{c}_i = \{c_{ij}\}_{j=1}^N, \ c_{ij} = \#\{y \in \mathcal{I} : d(x_i, y) \in [r_{j-1}, r_j)\}$$

where the $0 = r_0 < r_1 < \cdots < r_N$ is an increasing sequence of radii. For each cancer cell, the counts are from a volume constrained both by the size of the annulus and the environmental domain. Call this sequence of volumes $\mathbf{V}_i = \{V_{ij}\}_{j=1}^N$ so that V_{ij} represents the volume of the annulus centered at x_i at a distance within $[r_{j-1}, r_j)$ and within the simulated domain. Note: cancer cells in different parts of the domain will have different sequences of volumes because they have different proximities to the domain boundary.

Next, sum across all cancer cells these two types of vectors: sum the counts vectors and sum the volume vectors. Dividing these two vectors component-wise gives the penultimate value for computing cross-PCF values:

$$\tilde{\mathbf{g}} = \{\tilde{g}_j\}_{j=1}^N, \ \tilde{g}_j = \frac{\sum_i c_{ij}}{\sum_i V_{ij}}$$

Finally, these values are normalized by the average density of immune cells over the entire domain. Letting V represent the total volume of the domain, we then arrive at the final cross-PCF values:

$$\mathbf{g} = \{g_j\}_{j=1}^N, \ g_j = \frac{\tilde{g}_j}{(\#\mathcal{I})/V}$$

When combining cross-PCF values across replicates, we average the vectors corresponding to a particular time point across the replicates. The computation of these cross-PCF values used PairCorrelationFunction.jl v0.0.11 at https://github.com/drbergman-lab/PairCorrelationFunction.jl.

PhysiCell neighbors graph and analysis

PhysiCell labels two cells as neighbors if they physically overlap in the simulation. That is, if the distance between the two cell centers is less than the sum of their radii, they are neighbors. This information is output at each save point as an undirected graph with vertices representing cells and edges representing neighboring. Using this information, we can compute the connected components. A single connected component consists of all cells that can be reached starting from a single cell and following the neighbor edges of this graph. In PhysiCell, when a cell begins a death process, apoptosis or necrosis, it immediately is removed from all neighbors lists, so we exclude these from this analysis.

Supplementary Figures

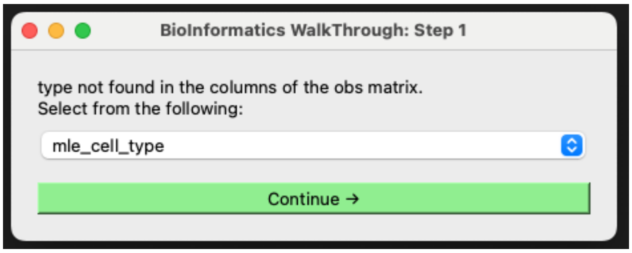

Fig. S1. Select the feature of the metadata containing cell type information.

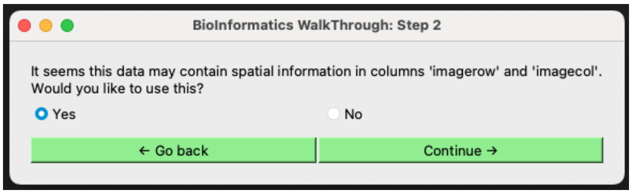

Fig. S2. If spatial data is detected, confirm the use of spatial data.

Competing interests

LZ receives grant support from Bristol-Myers Squibb, Merck, Astrazeneca, iTeos, Amgen, NovaRock, Inxmed, and Halozyme. LZ is a paid consultant/Advisory Board Member at Biosion, Alphamab, NovaRock, Ambrx, Akrevia/Xilio, QED, Tempus, Pfizer, Novagenesis, Snow Lake Capitals, Amberstone, Tavotek Lab, ClinicalTrial Options, LLC, and Mingruizhiyao. LZ holds shares at Amberstone, Alphamab, Cellaration, and Mingruizhiyao.

EMJ reports other support from Abmeta and Adventris, personal fees from Dragonfly, Neuvogen, Surge Tx, Mestag, HDTbio, and grants from Lustgarten, Genentech, BMS, NeoTx, and Break Through Cancer. EMJ is a founder of and holds equity in Adventris Pharmaceuticals. She also serves as a consultant to the entity.

EJF is on the Scientific Advisory of Resistance Bio/Viosera Therapeutics, a paid consultant for Merck and Mestag, and receives research funds from Abbvie Inc, Roche/Genetech, and Break Through Cancer outside the submitted work.

Author contributions statement

Conceptualization: DRB, PM, EJF. Data curation: DRB, DNS, LTK, RAA, LZ, EMJ, EJF. Formal analysis: DRB. Funding acquisition: DRB, AD, RH, LTK, RAA, LZ, EMJ, GSO, PM, EJF. Investigation: DRB, JJ, MN, MB. Methodology: DRB, JJ, MN, MB, HLR, AD, TLV, RH, GSO, PM, EJF. Project administration: DRB, PM, EJF. Resources: DRB, DNS, LTK, RAA, LZ, EMJ, EJF. Software: DRB, JJ, MN, MB. Supervision: DRB, GSO, PM, EJF. Validation: DRB, JJ, MN. Visualization: DRB. Writing-original draft: DRB, MN. Writing-review & editing: DRB, JJ, MN, HLR, AD, DNS, TLV, RH, LTK, EMJ, GSO, PM, EJF.

Acknowledgments

Funding was provided by P01CA247886 (to EMJ, LTK, EJF), U24CA284156 (EJF, PM, HLR, DRB, RH, GSO, AD), 1U01CA294548-01 (EJF, PM, DRB), the Lustgarten Foundation 'A Translational Convergence Program of Personalized Immunotherapy for Pancreatic Cancer Patients at Johns Hopkins' (EMJ, LTK, EJF, AD), GI SPORE P50CA062924 (EMJ, EJF, LTK), U01CA253403 (EJF, AD, EMJ), U54CA274371 (EJF), U01CA212007 (EJF, LTK), U54CA268083 (EMJ, EJF), R00NS122085 (GSO), U01CA284090 (GSO), T32CA153952

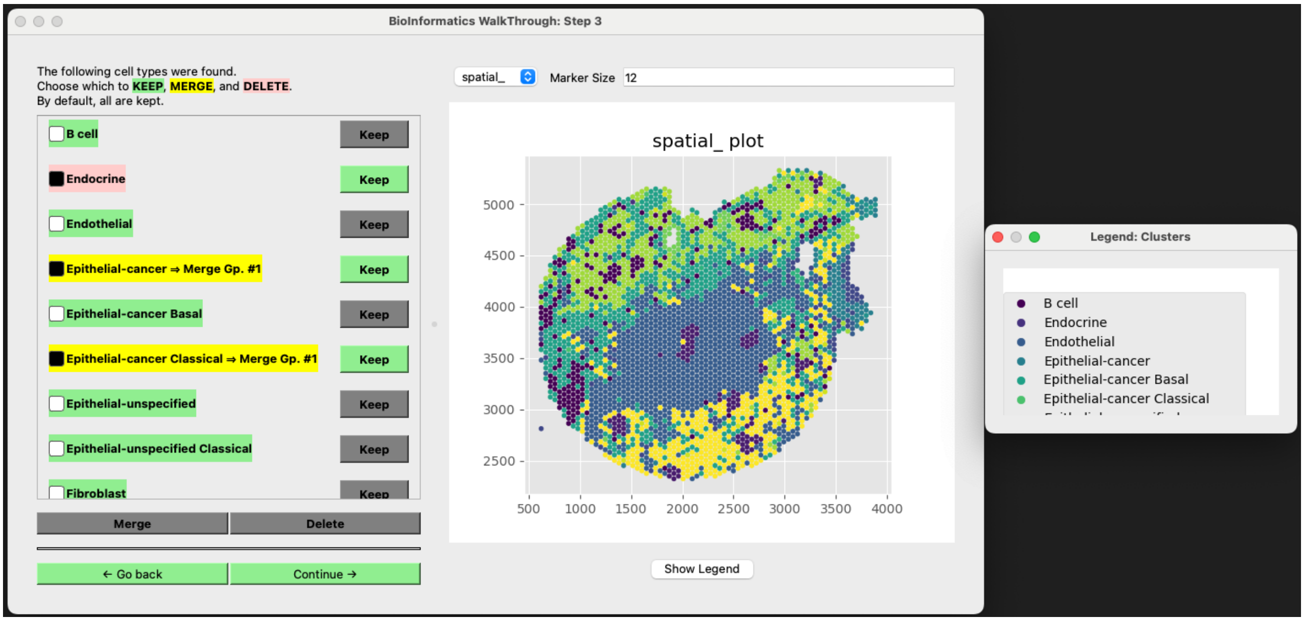

Fig. S3. Curate cell types found in the selected metadata column of Step 1, including merging of multiple cell types or deletion of cell types. In the right panel, visualize the data using any dimensionality reductions found (PCA, t-SNE, UMAP) or the spatial coordinates if found.

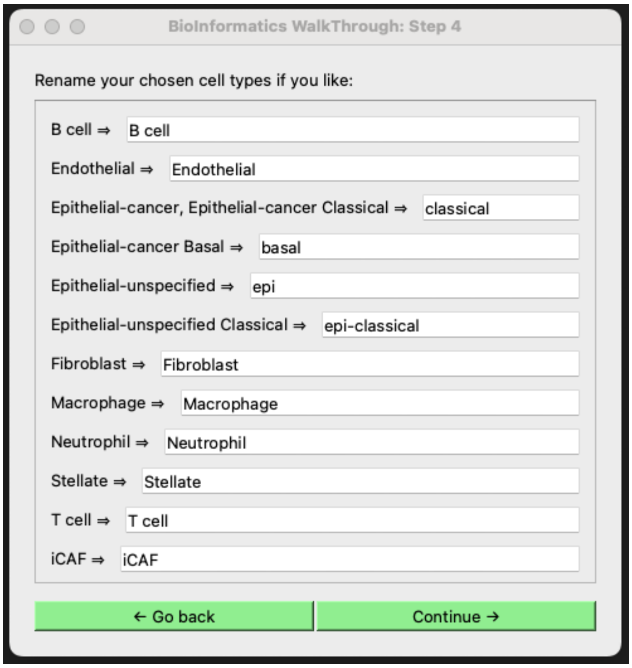

Fig. S4. Rename cell types, including merged groups of cell types, to match the cell type names in the agent-based model.

(DRB), NSF 1720625 (RH, PM), NSF 2303695 (RH, HR, PM), the National Foundation for Cancer Research (EJF), the Jayne Koskinas Ted Giovanis Foundation for Health and Policy (RH, HLR, PM), U01CA232137 (RH, PM), NSF 1818187 (RH, PM), Leidos Biomedical Research contract 75N91019D00024 (RH, HLR, PM), Maryland Cancer Moonshot Research Grant to the Johns Hopkins Medical Institutions (FY24) (AD, EJF), a Luddy Faculty Fellowship (HLR, PM), R01CA169702 (LZ), R01CA197296 (LZ), and P30CA006973 (LZ), NCI P30

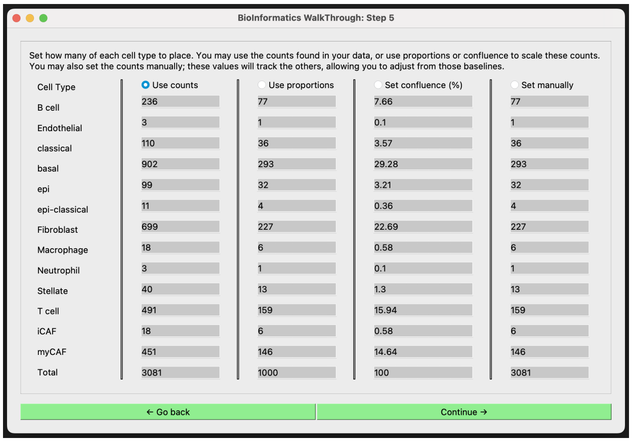

Fig. S5. If not using spatial data, set cell counts to use based on the data. `Use counts` uses the counts found in the data and cannot be changed.

CA054174 (LZ), Break Through Cancer (EJF, AD, LTK, EMJ, LZ), and the Maryland Cigarette Restitution Fund Research Grant to the Johns Hopkins Medical Institutions (FY24) (AD). This article was supported by funds through the National Cancer Institute – Cancer Center Support Grant (CCSG) - P30CA134274. The Jayne Koskinas Ted Giovanis Foundation for Health and Policy is a private foundation committed to critical funding of cancer research. The opinions, findings, conclusions or recommendations expressed in this material are those of the authors and not necessarily those of the Jayne Koskinas Ted Giovanis Foundation for Health and Policy or its respective directors, officers, or staff.

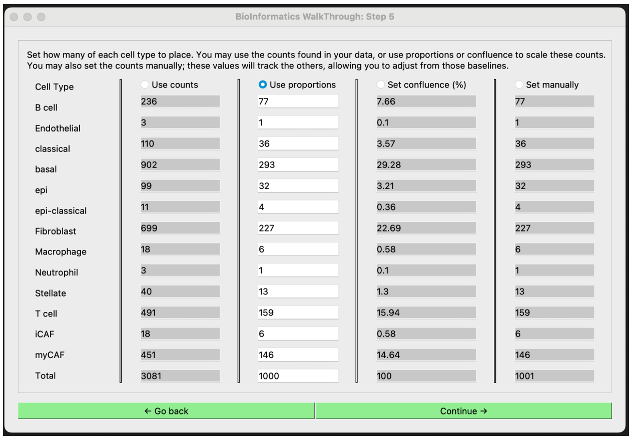

Fig. S6. If not using spatial data, set cell counts to use based on the data. `Use proportions` preserves the proportionalities of the cell types present in the `Use counts` column; changes to any entry trigger updates to all others to preserve these proportions up to rounding.

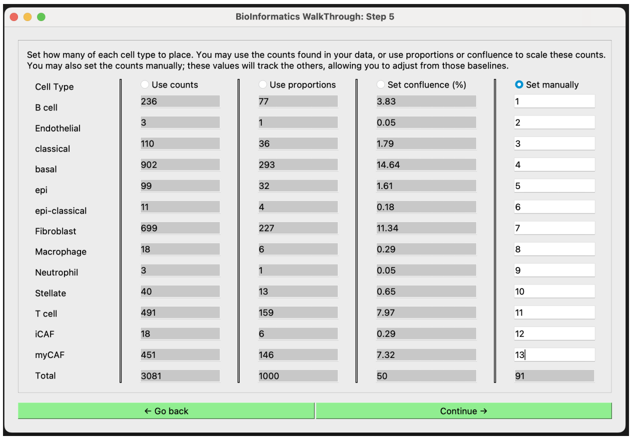

Fig. S8. If not using spatial data, set cell counts to use based on the data. `Set manually` sets the cell counts independent of the data entirely. The values in this column update to reflect the other columns when those are selected. In that way, users can adjust the numbers calculated in those columns as needed.

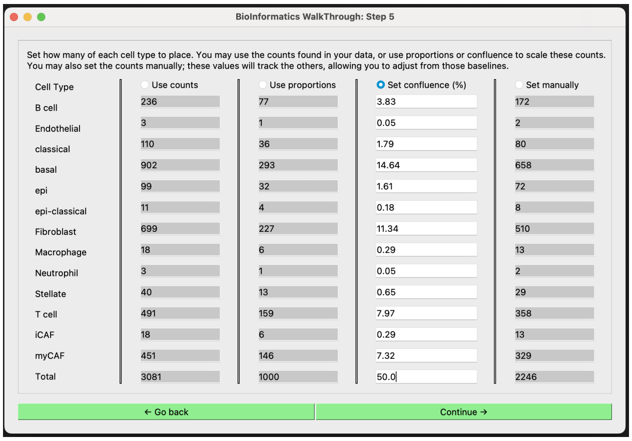

Fig. S7. If not using spatial data, set cell counts to use based on the data. `Set confluence (%)` behaves similarly to `Use proportions` but uses the cross-sectional area of the cell types (i.e., confluence)–using the volume of the cell type present in the configuration file loaded when Studio is launched; defaults to the default cell type volume if the cell type is not found–to set the amount of cells of each type.

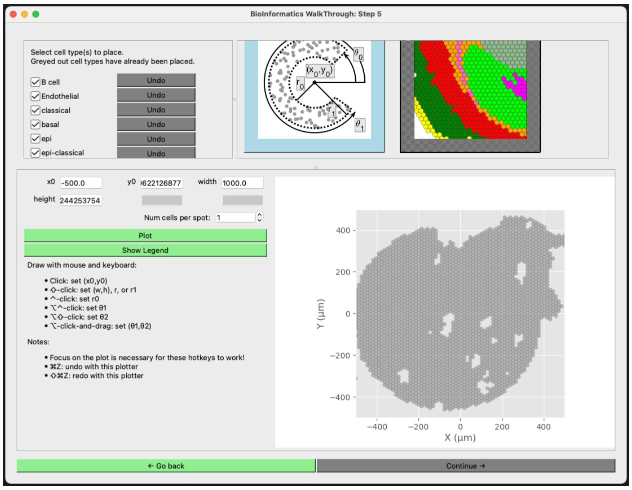

Fig. S9. If using spatial data, the `spatial` plotter is selected by default, which shows a dot at each cell location sized according to the cell volume. The spatial coordinates are extended to fill the simulation domain while preserving the aspect ratio. Multiple cells can be placed per spot using the `Num cells per spot` parameter in the bottom-left panel. The dots grow according to the total cross-sectional area of the cells placed randomly, uniformly within this area.

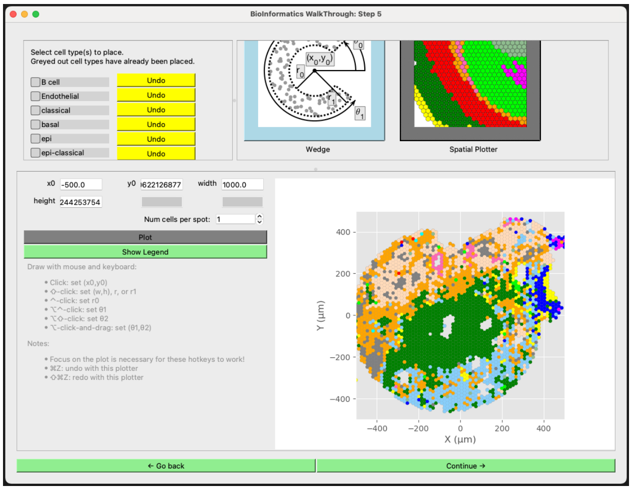

Fig. S10. The spatial data after plotting.

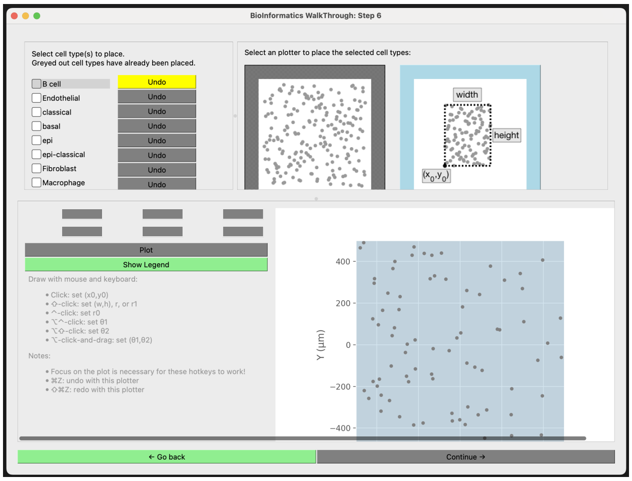

Fig. S11. Place the cells in the simulation domain. If not using spatial data, five plotters are available to place the cells: `everywhere`, `rectangle`, `disc`, `annulus`, and `wedge`. By selecting a plotter, the parameters in the bottom-left panel update and can be set manually or using mouse-and-keyboard commands listed in the bottom-left panel. A shaded region (the entire domain here using the `everywhere` plotter; see Fig. S12-S15 for other shapes) updates in real time to show where the cells would be placed. For each plotter, a history of parameter values is tracked that can be accessed via the standard operating system (OS) hotkeys, e.g. Cmd+Z and Cmd+Shift+Z on a Mac. After selecting one or more cell types (top-left panel), the `Plot` button is enabled and clicking the button places the number of cell types from the previous step uniformly in the intersection of the shaded region and the domain environment, i.e., exactly what can be seen of the region in the figure. At that point, the plotted cell types are disabled in the top-left panel and their `Undo` buttons are enabled. After all cell types are plotted, the `Continue` button is enabled. Here, the `everywhere` plotter is shown along with the `B cell` cells placed randomly, uniformly within.

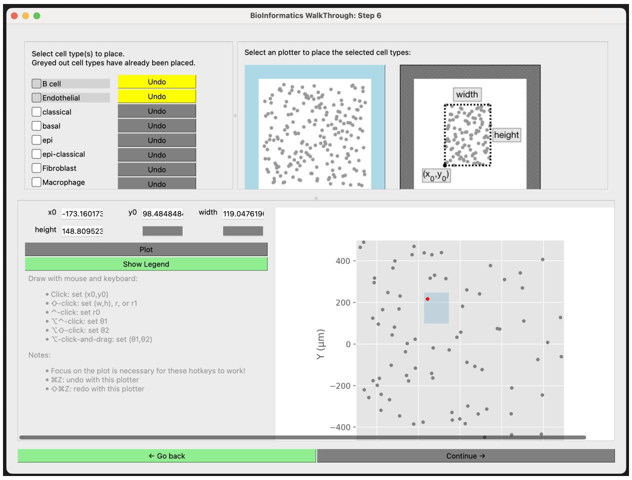

Fig. S12. The `rectangle` plotter with the (one) `endothelial` cells placed randomly, uniformly within.

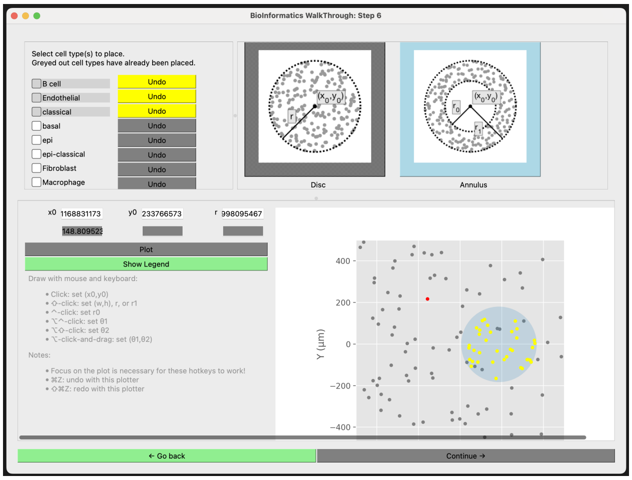

Fig. S13. The `disc` plotter with the yellow `classical` cells placed randomly, uniformly within.

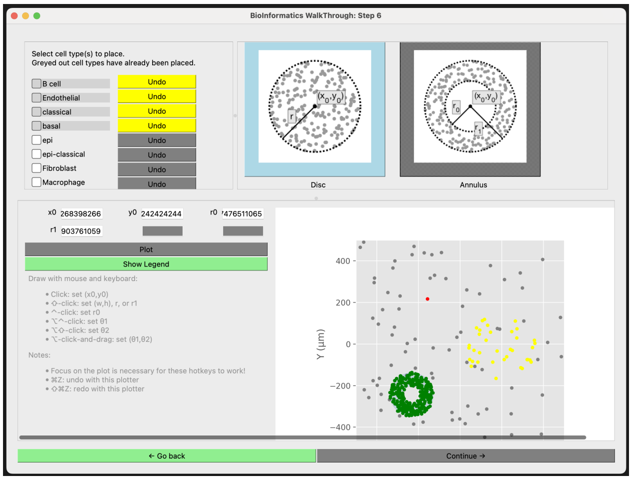

Fig. S14. The `annulus` plotter with the green `basal` cells placed randomly, uniformly within.

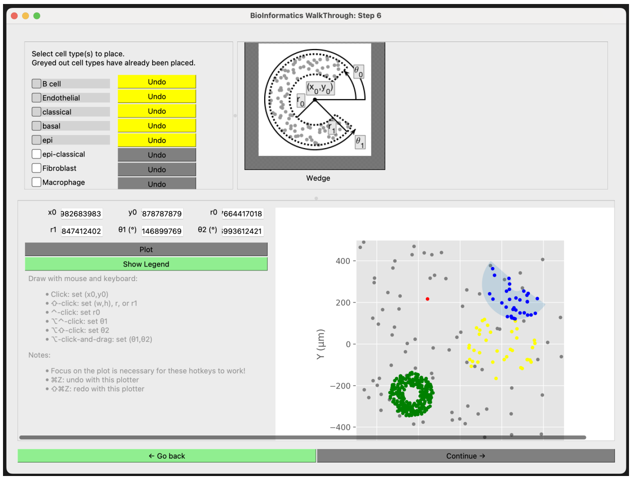

Fig. S15. The `wedge` plotter with the blue `epi` cells placed randomly, uniformly within.

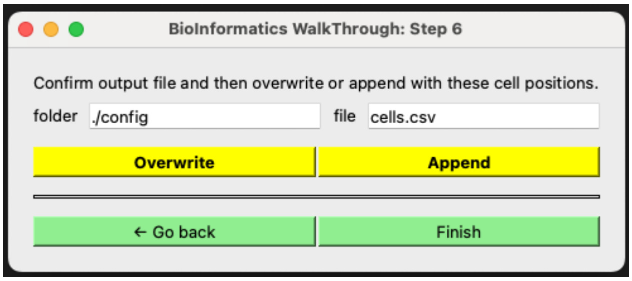

Fig. S16. Write the positions and cell types to the PhysiCell-readable format, either overwriting a previous cell initialization file or appending to one already present.

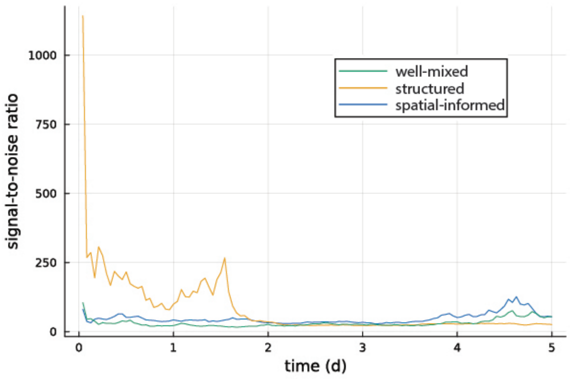

Fig. S17. Signal-to-noise ratio of cancer cell count over time for each model initialization.

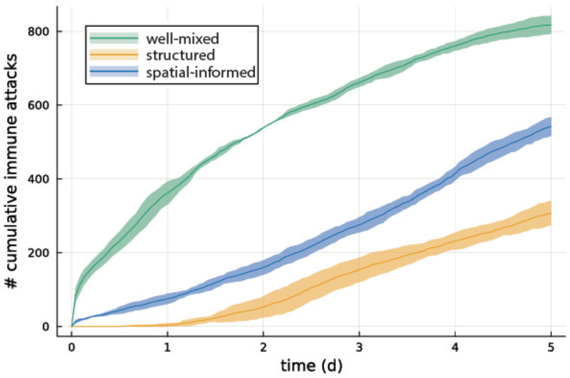

Fig. S18. Cumulative immune attacks over time for each model initialization.

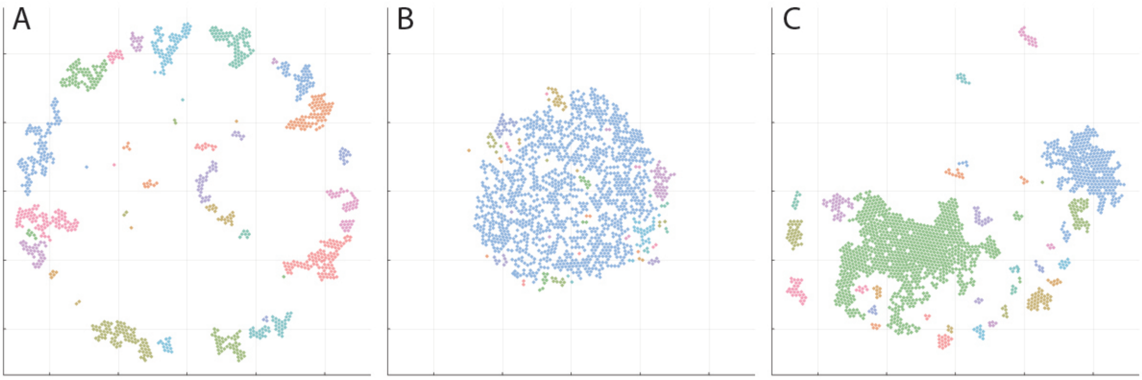

Fig. S19. Connected components of cancer cells at final time point for the well-mixed (A), structured (B), and spatial-informed (C) initializations. Compare with Figure 2D-F. Each cell is colored according to the connected component to which it belongs.
